# Structure of 311 service requests as a signature of urban location

**DOI:** 10.1371/journal.pone.0186314

**Published:** 2017-10-17

**Authors:** Lingjing Wang, Cheng Qian, Philipp Kats, Constantine Kontokosta, Stanislav Sobolevsky

**Affiliations:** 1 Center for Urban Science and Progress, New York University, Brooklyn, New York, United States of America; 2 Tandon School of Engineering, New York University, Brooklyn, New York, United States of America; 3 Kazan Federal University, Kazan, Russia; 4 Department of Civil and Urban Engineering, New York University, Brooklyn, New York, United States of America; 5 Senseable City Laboratory, Massachusetts Institute Of Technology, Cambridge, Massachusetts, United States of America; 6 Institute Of Design And Urban Studies of The Saint-Petersburg National Research University Of Information Technologies, Mechanics And Optics, Saint-Petersburg, Russia; Peking University, CHINA

## Abstract

While urban systems demonstrate high spatial heterogeneity, many urban planning, economic and political decisions heavily rely on a deep understanding of local neighborhood contexts. We show that the structure of 311 Service Requests enables one possible way of building a unique signature of the local urban context, thus being able to serve as a low-cost decision support tool for urban stakeholders. Considering examples of New York City, Boston and Chicago, we demonstrate how 311 Service Requests recorded and categorized by type in each neighborhood can be utilized to generate a meaningful classification of locations across the city, based on distinctive socioeconomic profiles. Moreover, the 311-based classification of urban neighborhoods can present sufficient information to model various socioeconomic features. Finally, we show that these characteristics are capable of predicting future trends in comparative local real estate prices. We demonstrate 311 Service Requests data can be used to monitor and predict socioeconomic performance of urban neighborhoods, allowing urban stakeholders to quantify the impacts of their interventions.

## 1 Introduction

Cities can be seen as a complex system composed of multiple layers of activity and interactions across various urban domains; therefore, discovering a parsimonious description of urban function is quite difficult [1–4]. However, urban planners, policy makers and other types of urban stakeholders, including businesses and investors, could benefit from an intuitive proxy of neighborhood conditions across the city and over time [5–7]. At the same time, such simple indicators could provide not only valuable information to support urban decision-making, but also to accelerate the scalability of successful approaches and practices across different neighborhood and cities, as urban scaling patterns have become an increasing topic of interest [8–12]. As the volume and heterogeneity of urban data have increased, machine learning has become a viable tool for enhancing our knowledge of urban space and in developing predictive analytics to inform city management and policy [13–16].

The non-trivial challenge is to identify a consistent, quantifiable metric that provides comprehensive insights across multiple layers of urban operations and planning [17] and to locate readily-available data to support its implementation across a range of cities. Fortunately, urban data collected by various agencies and companies provide an opportunity to respond to this challenge [18, 19]. In the age of ubiquitous digital media, numerous aspects of human activity are being analyzed by means of their digital footprints, such as mobile call records [20–25], vehicle GPS traces [26], bank card transactions [27–29], payment patterns [30–32], smart card usage [33–35], or social media activity [36–40]. Such data sets have been successfully applied to investigate urban [41] and regional structure [42, 43], land use [44, 45], financial activities [46], mobility [47, 48], or well-being [49, 50].

However, one of the major limitations to widespread adoption of such analytics in the practice of urban management and planning is the extreme heterogeneity of the data coverage: different types of data are available for different areas and periods of time, which undermine efforts to develop universal and reliable analytic approaches. Privacy considerations are another significant issue that create additional practical and legal obstacles, restricting data access and preventing their use out of a concern for confidentiality [51–54].

Increasingly, cities are introducing systems to collect service requests and complaints from their citizens. These data, commonly referred to as 311 requests, reflect a wide range of concerns raised by city residents and visitors, offering a unique indicator of local urban function, condition, and service level. In many cities, 311 requests are publicly available through city-managed open data platforms as part of a broader movement in local government to increase transparency and good governance [55–57]. Although potentially biased by the self-reported nature of the requests and complaints, these data provide a comparable measure of perceived local quality of life across space and time.

In this article, we develop a method for classifying urban locations based on the categorical and temporal structure of 311 Service Requests for a given neighborhood, exploring whether these spatio-temporal patterns can reveal characteristic signatures of the area. For New York, Boston, and Chicago, we present applications of this new urban classifier for predicting socioeconomic and demographic characteristics of a neighborhood and estimating the economic performance and well-being of a defined spatial agglomeration. The paper begins with a discussion of the data and methodology, followed by specific use cases relating to demographics and real estate values, and concluding with opportunities for future research.

## 2 Materials and resources

### 2.1 The 311 data

311 service request and complaint data are being collected across more than 30 cities in the United States, including New York, Boston and Chicago. The data for those three cities is publicly available [58–60]. Through the 311 system, local government agencies offer non-emergency services to residents, visitors and businesses and respond to reported service disruptions, unsafe conditions, or quality-of-life disturbances. While requests are collected through multiple sources, including text messages, web page, and dedicated mobile applications, vast majority of them comes through the phone calls. These 311 service requests and complaints cover a wide range of concerns, including, but not limited to, noise, building heat outages, rodent sightings, etc. Thus, these data serves as an extremely useful resource in understanding the delivery of critical city services and neighborhood conditions.

We explore the 311 datasets from New York, Chicago, and Boston as major urban centers where 311 systems are in place and commonly used. We consider a time frame between 2012 and 2015 during which the data are available for all three cities selected. In Table 1, we provide descriptive statistics of the data. Note that the number of total requests has been increasing from 2012 to 2015 in each city. Conceivably, the number of requests in New York City (which now approaches 2 million per year) is higher than the others because of its population size (over 8.5 million). Boston municipality (central part of the city for which the 311 data is collected with a population around 600.000) has a substantially smaller number of requests compared to the city of Chicago (around 2.7 million people) and of course much smaller than NYC) Unfortunately, each city uses a different complaint/request coding convention, thus there is little consistency in the classification of particular complaint types. This fact raises certain difficulties for analysis between cities, a common challenge in comparative urban analytics given the lack of data standardization. For example, in 2015, New York City’s 311 data are categorized into 182 types, where Chicago has only 12. Even within a particular city, request categories are subject to change over time, especially in NYC where only approximately 70% of the entire service request activity belong to common categories present in all four years. Additional adjustments are needed to re-classify complaint types into standardized categories across the different cities and over the time period of the analysis.

**Table 1:** General properties of the 311 data for NYC, Chicago and Boston.

Year	New York City		
	Total Requests	Requests Categories	Share of common categories’ activity
2012	1414392	165	0.69
2013	1431729	162	0.69
2014	1654913	179	0.73
2015	1806560	182	0.73
Year	Chicago		
	Total Requests	Requests Types	Share of common categories’ activity
2012	478532	13	0.85
2013	507956	14	0.82
2014	515258	14	0.82
2015	568576	12	0.9
Year	Boston		
	Total Requests	Requests Types	Share of common categories’ activity
2012	92855	155	1
2013	112727	165	0.99
2014	112785	183	0.96
2015	161498	180	0.83

The original data set provided by 311 Services contains one record for each customer’s call. For most cities, these records include information such as: service request type, service request open/close time and date and location(longitude and latitude). With that information, we can aggregate the 311 service requests and group by type for any given time period and area(census tract area, block groups, zipcode).

### 2.2 Demographic and socioeconomic data

As we are attempting to use 311 data as a proxy for the socioeconomic characteristics and real estate values of urban neighborhoods, ground-truth data are needed to train and validate our models. For socioeconomic and demographic features, we use data from the U.S. Census 2014 American Community Survey (ACS). For real estate values, we collect housing price data from the online real estate listing site Zillow. Both are described below.

#### 2.2.1 2014 census data

The 2014 ACS contains survey data on a number of socioeconomic and demographic variables, at the spatial aggregation of the Census Blocks. For this analysis, we have selected common features representing important phenomena in population diversity, education, and income and employment. For example, our selection covers the number of population in the following categories: “Non-Hispanic White”, “African-American”, “Asian”, “High school degree”, “College degree”, “Graduate degree”, “Uninsured ratio”, “Unemployment ratio”, “Poverty ratio”, and mean for “Income (all)”, “Income of No Family”, “Income of Families” and “Income of Households”.

One important consideration is the level of spatial aggregation for this analysis. Having considered zip code, census tract and census block groups, we decided to proceed with census tracts providing the best trade-off between spatial granularity, in terms of having a sufficient number of sub-areas within each city, and having a statistically significant sample of 311 complaints for each areal unit. In Boston and Chicago, there are too few zipcodes within in each city to create a useful sample, and there is not a significant density of 311 complaints at the census block group level (please refer to S4 Text for details). In addition, given the survey methodology of the ACS data, census block groups data include non-trivial margins-of-error for each variable.

#### 2.2.2 Zillow housing price

One important indicator of local economic conditions is housing prices [61]. We utilize Zillow housing price data that contain monthly average residential real estate sales prices by zip code. Although housing prices are a lagging indicator of neighborhood economic strength, since recorded sales occur as much as two to more than six months after a contract is signed, we use these values as one of the targets for our 311 predictions. Our spatial level of analysis will be the zipcode, rather than census tracts, given the coverage area of the Zillow aggregate data.

#### 2.2.3 Normalization method and some notations

In order to better compare various areas, the census data need to be normalized. Take income per capita and population with bachelor degree for example. Firstly, these two features have different measurement units (dollars versus number of people). Secondly, this number can be affected by the area’s total population. For an area with high population, there should be a higher possibility to have higher population with bachelor degree. Therefore, the normalization process is important in order to compare different features and different areas with heterogeneous population. For our analysis, we normalize our census tract data set in the following way.

Let *p*_*i*_ be the total population in census tract, while *v*_*i*_ denotes one feature recorded in the same census tract *i*, for example, “the total population who holds graduate degree in census tract *i*”. Next, we normalize it by defining

$$\begin{array}{c} y_{i}=\frac{v_{i}p_{i}}{\sum_{j\in \Omega }v_{j}p_{j}} \end{array}$$

We define Ω as a set of all census tracts in New York City.

In section 2, we use 311 complaint frequency categorized by census tracts to cluster and investigate the difference in local socioeconomic features *y*. In section 3 we use machine learning regression models to predict these features *y* using normalized 311 data.

## 3 Classification based on 311 service categories

In order to get initial insights on the usage of 311 service across the considered cities, we define for each census tract area, a 311 pattern signature—a vector of the relative frequencies of 311 requests of different types. This signature is supposed to characterize the unique way people use 311 service in the given neighborhood, showing which particular concerns are the most important ones for the local community. Each *t*-th component of the signature vector will be the frequency of each category of requests *t* among all the requests made with a given area *a*. Specifically, let the total number of service requests of each type *t* within an area *a* be *s*(*a*, *t*) and let *s*(*a*) = ∑_*t*_ *s*(*a*, *t*) be the total number of service requests in the area *a*. Then a vector *S*(*a*) = (*s*(*a*, *t*)/*s*(*a*), *t* = 1..*T*), where *T* is the total number of service request types, serves as a signature of the location’s aggregated 311 service request behavior. The vector *S* highlights the primary reasons for service requests or complaints in the specific area, allowing for straightforward comparison across tracts and cities.

Signatures *S*(*a*) serve as unique characteristics of each location *a*, and we would expect similar spatio-temporal patterns to emerge in 311 service requests across a city or cities. Our hypothesis here is that these similarities also suggest similarities in the socioeconomic characteristics of the areas. In order to explore this further, we apply a k-mean clustering approach to the set of multi-dimensional vectors *S*(*a*). In order to ensure we get an optimal clustering we run the algorithm 100 times, selecting the best solution in terms of cumulative square sum of distances from centroids. A comparison with an alternative clustering approach DBSCAN [62] able to handle the outliers is provided in the S6 Text. Also while k-means clustering deals exclusively with the 311 patterns without any spatial considerations, an impact of additional spatial regularization is considered in S7 Text.

One crucial step in this approach is to pick up an appropriate number of clusters to consider. For that purpose we have evaluated the clustering model with both Silhouette method and Elbow method. While different methods give a slightly different optimal number of clusters for the cities in our sample, in most cases it is within a range of two to four clusters. Given the socioeconomic diversity across neighborhoods in the selected cities, we determine that a minimum of four clusters is an appropriate value. Readers can find more details in S1 Text.

**Fig 1:**
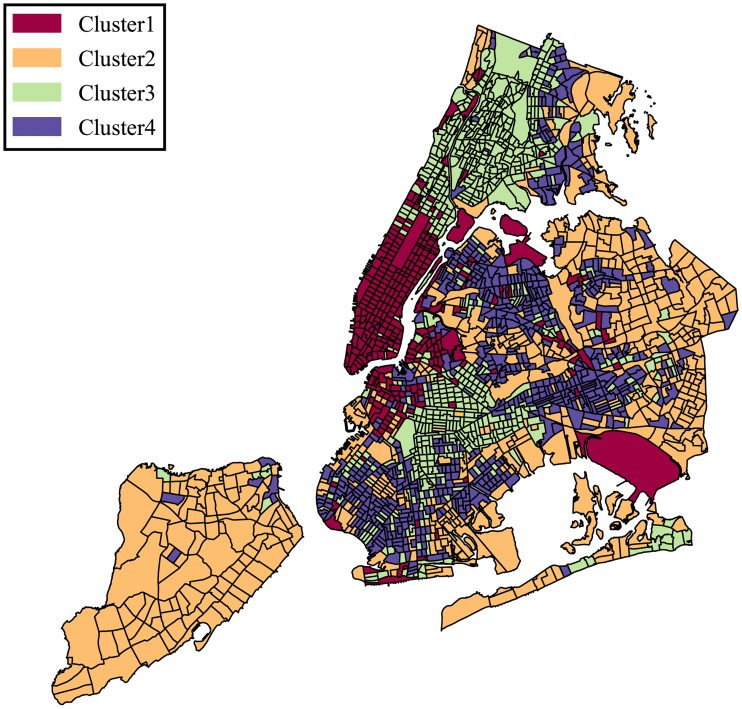
Classification of urban locations based on the categorical structure of the 311 requests.

We consider NYC first. In Fig 1, we see below with approximate 2000 census tracts divided into four clusters based on our clustering results. Midtown Manhattan, downtown Brooklyn and several outliers such as JFK and LGA airports belong to cluster 1; Staten Island and eastern Brooklyn/Queens constitute cluster 2; Northern Manhattan, the Bronx, and central Brooklyn are included in cluster 3; and Southern Brooklyn, Flushing and some eastern parts of Bronx comprise cluster 4.

**Fig 2:**
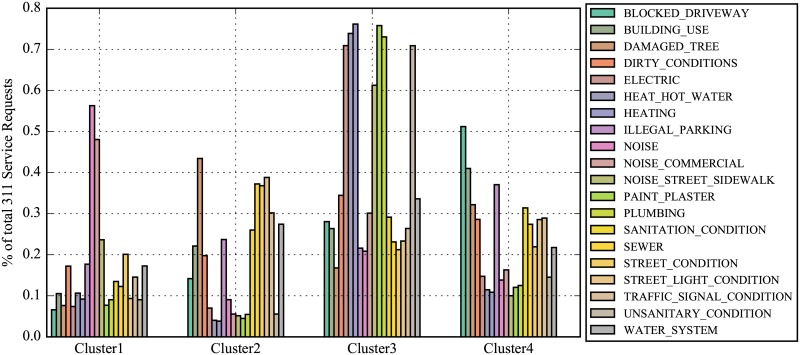
Patterns of 311 activity within clusters: Top 20 service request categories and their frequency distribution among clusters.

In order to evaluate how different each cluster is with respect to the nature of 311 service requests, (see Fig 2) we present the distribution of top service requests over the four clusters. We observe clear variation in this distribution. For example, complaints/requests within cluster 1 more often report noise concerns than others, cluster 2 experiences more issues relating to residential heating, cluster 3 has the highest relative complaints about blocked driveways, while cluster 4 reports concerns about street conditions. Clusters 2 and 4 are perhaps the most similar ones in terms of their profiles, they still present considerable differences, such as blocked driveway complain intensity. Indeed those two clusters represent different parts of Brooklyn-Queens area, while cluster 2 is more residential, which perfectly explains why blocked driveways are that much of a concern.

**Fig 3:**
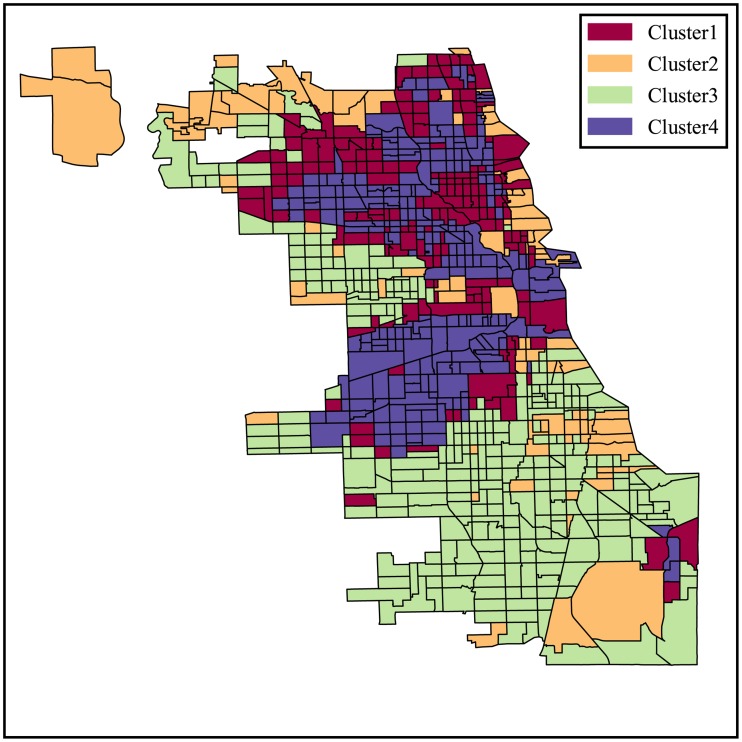
Classification of urban locations based on the categorical structure of the 311 service requests for Chicago.

**Fig 4:**
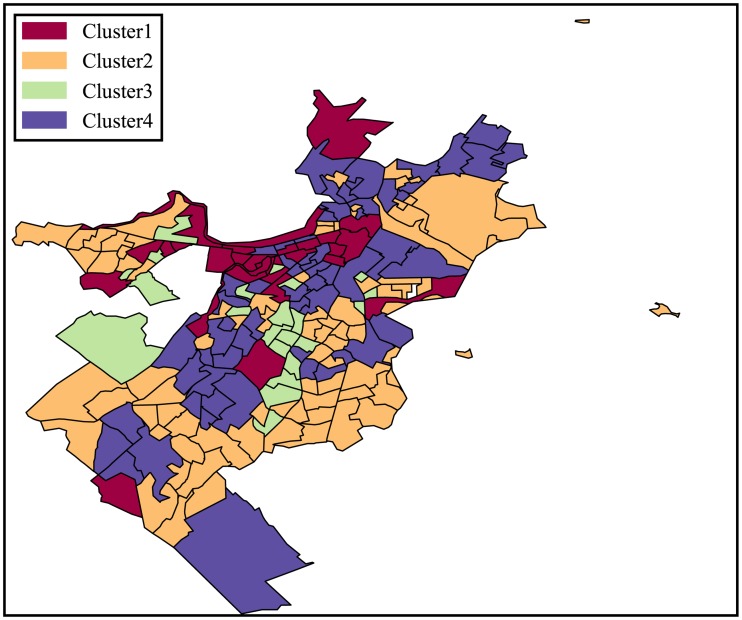
Classification of urban locations based on the categorical structure of the 311 service requests for Boston.

Similarly, we repeat the same clustering process for Chicago and Boston and the clustering results for census tracts in those cities are shown in Figs 3 and 4.

An alternative 311-based clustering approach using timelines of the 311 activity rather than the categories of 311 requests is considered in S2 Text.

## 4 Socioeconomic features among clusters

Given the knowledge of the local spatial contexts for the analyzed cities, the clusters that emerge make certain intuitive sense. However, in order to quantitatively address the hypothesis formulated in the previous section—that similarities in local 311 service request signatures also imply similarities in the socioeconomic profiles of those areas—here we summarize and analyze the socioeconomic characteristics for each of the discovered clusters.

**Fig 5:**
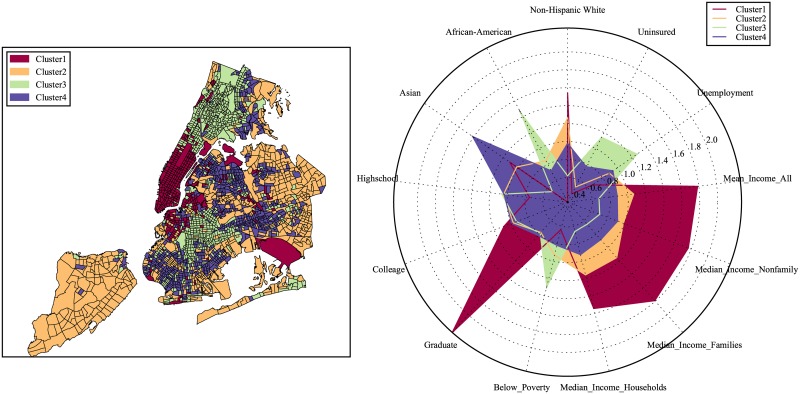
Comparison of the average level of socioeconomic features among clusters in New York.

**Fig 6:**
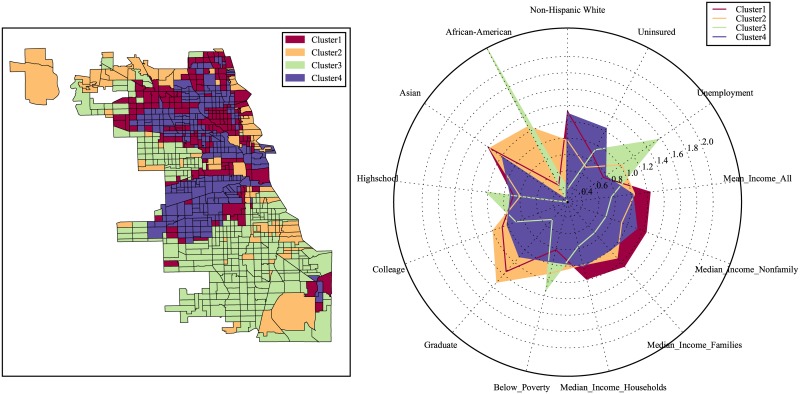
Comparison of the average level of socioeconomic features among clusters in Chicago.

**Fig 7:**
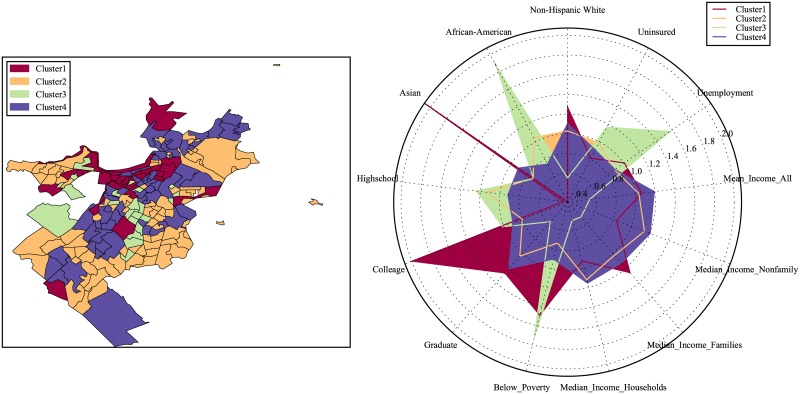
Comparison of the average level of socioeconomic features among clusters in Boston.

Recall that thus far the clustering results are obtained based on the 311 service requests frequency alone with no socioeconomic information considered. Next we summarize the socioeconomic features such as the levels of income, education, unemployment, medical insurance as well as racial decomposition, and compare the normalized mean level for each feature in each of the considered clusters. The results for our three cities are presented in the radar plots in Figs 5–7. From the output, we can see that the socioeconomic features among the defined clusters are quite distinctive.

Take NYC for example:

- Education and Income: People with higher levels of education (with graduate degree and above) are found in cluster 1, which, as expected, also has highest income level. Cluster 3 appears to show the opposite results.
- Racial diversity: There are above average concentrations of Non-Hispanic Whites living in clusters 1 and 2, of Asian origin in cluster 4, and African-American populations in cluster 3.

Similarly we have (for both Chicago and Boston):

- Cluster 1 has the highest income and education level, while cluster 3 is the lowest.
- Cluster 2 is predominantly Asian and African-Americans, while Non-Hispanic Whites tend to live in clusters 1 and 4.

The observations above provide some evidence for our hypothesis, revealing links between socioeconomic features and 311 service request data structure. Indeed, while the clustering is performed based on the 311 data alone, the socioeconomic features happen to be quite distinctive among the clusters. Of course this only reveals the existence of a certain relation in principle, which might not be that practical. However this gives rise to another hypothesis—can one use 311 service request data to actually model socioeconomic features at the local scale?

## 5 Modeling the socioeconomic features

We find that 311 service request signatures allow the city to be divided into clusters based on distinctive patterns of socioeconomic characteristics. Following this, we explore whether 311 service requests can be used to model these socioeconomic patterns. Such a model could be useful as socioeconomic data are often unavailable or inconsistent at a given spatio-temporal scale, and therefore having a proxy derived from a model based on regularly-updated open data could have considerable potential for city operations and neighborhood planning.

We train regression models in order to estimate the selected socioeconomic features described in subsection 1.2.1, using relative frequencies of 311 service requests of each type in each census tract as predictors. This way the service request frequencies *s*(*a*, *t*)/*s*(*a*) (components of the signature vectors as used in the section 2, showing how often a service request within a given area *a* belongs to a given category *t*) constitute our feature space, including 179 different features (one per each request type *t*) in the case of NYC, across 2000 census tracts following the data cleaning/filtering process.

We consider six target variables to model including income per capita, percentage of residents with a graduate degree, percentage of unemployed residents, percentage of residents living below the poverty level, as well as demographic characteristics including percentage of Non-Hispanic White and African-American populations.

The objective of the modeling is to use partial information about the target variables defined in a certain part of the city to train the model so that it can explain the target variables over the rest of the city.

For the purpose of a comprehensive model evaluation we use a cross-validation procedure, training the model over different subsets of the data sample and evaluating its performance over the rest of the data (how well the modeled values of target variable correspond to the known values). We try several models including Lasso [63], Neural Networks with regularization (NN) [64–66], Random Forests Regression (RF) [67] and Extra Trees Regression (ETR) [68, 69].

For each model, we treat the different set of hyper parameters as different models. For Neural Networks, we try 5, 10, 20, 40 hidden unites and for each hidden unit, we try penalization lambda for 0.0005, 0.005, 0.05, 0.5. As to the learning process, we use mini batch size 20 and we use the following learning rate and epochs: (0.1,100), (0.05,200), (0.01,500), (0.005,1000). For RF and ETR, we use 500 trees(since increasing trees does not help) and try maximum leaf nodes: 10, 20, 30, … 100. In total we have 64 sets of hyper parameters for NN and 10 for RF and 10 for ERF.

More details on the model selection process is presented in S3 Text. We select the final model with suitable hyper parameters with the help of cross-validation. We divide the data set into training and testing set by the ratio 7:3. As described above, we have 84 different models. For each model, we randomly divide our training set into training and validation sets and train the model on the training set and report the R-squared on the validation set. We repeat this process 20 times for each model and get the average R-squared. We pick the best model and use it for prediction on the test set. Finally, we report the corresponding out-of-sample R-squared in Table 2. Generally speaking, RF and ETR usually give us best performance based on R-squared.

**Table 2:** Best 311-based model performance for modeling socio-economic features in different cities.

City	White/European	Afro-American	Graduate Degree	Income per cap	Below Poverty	Unemployment
NYC	0.54	0.50	0.48	0.70	0.44	0.26
Chicago	0.76	0.85	0.45	0.55	0.52	0.65
Boston	0.54	0.68	0.26	0.62	0.63	0.36

We consider this modeling result important because:

- it indicates that a relationship exists between 311 request signature and the local socioeconomic features of each area;
- it enables possible prediction and estimation of other local socioeconomic features by using 311 requests data, particularly those features for which data are collected at low temporal frequency, such as Census data; and,
- it can be easily scaled by geographic aggregation for various research, operational, or planning purposes.

For all 3 cities the data indicates spatial autocorrelation in both—311 patterns and target socio-economic quantities. Most of the 311 request categories demonstrate modest spatial autocorrelation, while some show a more substantial one; average values are reported in Table 3. Distributions of autocorrelation values for each category could be found in S3 Text. Thus, it seems reasonable to use spatial information for the modeling purposes. First, we check if spatial correlation occurs for the residuals for the existing models. Indeed, as displayed in Table 4, there is a significant correlation for most of the features. As those socio-economic features demonstrate high spatial autocorrelation, accounting for it could help further improving the models, at least when the entire neighborhood information is available. Next, we apply Spatial Lag model [70] to see how much it could help improving the accuracy. While performance vary across the features, this model works well for some of them, such as Median Income, Percentage of White/European, African-American and Asian population as reported in the Table 4. we also tried Spatial Error model but, as presented in Table 5, Spatial Lag largely outperforms it, so we stick to the best model going forward.

**Table 3:** Average spatial autocorrelation in the relative amounts of various categories of 311 service requests.

City	Avg. Autocorrelation	St.Dev.
NYC	0.19	0.175
Chicago	0.06	0.09
Boston	0.43	0.126

**Table 4:** Spatial autocorrelation for 311-based socio-economic model errors (Moran’s I).

City	Asian	Afro-American	Graduate Degree	Income per cap.
NYC	0.3	0.23	0.14	0.28
Chicago	0.3	0.23	0.14	0.28
Boston	0.2	0.41	0.26	0.18

**Table 5:** Spatial regression helping to improve OLS performance (In-Sample R-squared) for New York.

City	Asian	Afro-American	Graduate Degree	Income per cap.
OLS	0.42	0.58	0.49	0.69
Spatial Lag	0.74	0.84	0.65	0.73

While we see that spatial regression might improve the model in-sample performance, using it for predictive modeling is more problematic as it would require to learn the spatial correlation from the training data, which then need to span all over the city; this is rarely the case in practical applications. In any case the purpose of this study is not to build applicable predictive models but to show the modeling and/or predictive power of the 311 service requests, so that one can consider them for inclusion into the comprehensive applied models. This way we do not necessary aim for the paramount accuracy nor for a detailed modeling approach, including spatial regression techniques. Those are largely subject of the further study when 311-data will be incorporated into actual practical models together with all other available data sets.

## 6 Prediction of the real estate prices

Following our previous analysis, we attempt to understand the practical applicability of the prediction models. Although the findings above once again highlight a strong relation between 311 service request data and socioeconomic context of urban locations, this by itself has limited practical implications except for filling gaps in the data availability. In this section we show that 311 service request data could be also used to predict future socioeconomic variations, which may have more important practical implications for urban analytics.

As an example, consider the annual average sale price of housing per square foot in different neighborhoods of NYC as the target variable for our prediction. Our housing price is reported by Zillow at the zip code level; therefore, we rescale our 311 service request frequencies to this spatial aggregation.

To match available housing price data, we only include those 311 service categories that were recorded consistently between 2012 and 2015. New York City has 145 of such categories, covering about 70 percent of total service requests.

The target variable is updated annually and is available for each year from 2012 to 2015. The Zillow data cover 112 of the 145 zip codes in New York City where the density and frequency of 311 requests is sufficient to satisfy the filtering procedure described in the Data section. Thus, our sample for this prediction is based on data from 112 zipcodes.

We do not attempt to predict the absolute level of prices, but changes over time relative to the NYC mean. Our output therefore indicates how much more (less) expensive the housing price in a given zip code area is going to be compared to the average relative increase (decrease) in housing prices across NYC from the previous year. This way we define a new log-scale target variable *Y*^*t*^(*z*) in year *t* as

$$\begin{array}{c} Y^{t}\left(z\right)=log\left(P^{t}\left(z\right)/P_{mean}^{t}\right) \end{array}$$

where *P*^*t*^(*z*) is the average price per square foot in zip code *z* during the year *t*, while $P_{mean}^{t}$ is the average price per square foot across the entire city during the year *t*, estimated as the mean of *P*^*t*^(*z*) for all the locations *z* weighted by residential population of the locations used as a proxy for the locations’ size.

We begin by modeling the output variable *Y*^2015^. We train the model using 2012 and 2013 data (both—features and output variable) over the entire NYC and use 2014 data for tuning hyper-parameters, then apply it to 2015 using the features defined based on 2015 service requests. To reiterate, the feature space as before consists of the relative service requests frequencies *s*(*a*, *t*)/*s*(*a*), but now including only 145 categories of service requests, while the number of observations is 112 zip codes.

We subsequently train four different machine learning regression models as before: Lasso [63], Neural Networks with regularization (NN) [64–66], Random Forests Regression (RF) [67] and Extra Trees Regression (ETR) [68, 69].

The results are reported in Table 6 (we also include Boston and Chicago here just for comparison, although the number of zip codes in these cities is much smaller and thus the model becomes less significant).

**Table 6:** R-squared.

Model;	NYC		Chicago		Boston	
	In	Out	In	Out	In	Out
Lasso	.68	.49	.76	.57	.64	.38
NN(Regularized)	.84	.70	.82	.65	.84	.68
RF	.96	.78	.97	.81	.98	.79
ETR	.97	.79	.98	.90	.98	.83

As one can see from the Table 6, we achieve reasonable predictive power, especially with RF and ETR approaching *R*^2^ values of 0.80 for all three cities.

However, note that modeling housing prices in 2015 is not our objective here, since a simplified model *Y*^2015^ = *Y*^2014^ would achieve better results given the serial correlation in the time series and the relatively small year-to-year variation in price levels. Instead, we rather focus on the model’s ability to predict the magnitude and direction of those fluctuations, forecasting price trends at the zip code level.

Let $Y_{P}^{t}\left(z\right)$ be the predicted value of *Y*^*t*^(*z*). We define *D*(*z*) = *Y*^2015^(*z*) − *Y*^2014^(*z*) as the actual tendency of relative real estate prices in the zip code *z* and $D_{P}\left(z\right)=Y_{P}^{2015}-Y_{P}^{2014}$ as the predicted tendency of comparative housing price.

We classify the 112 zip codes of NYC into three groups based on the predicted tendency strength $D_{P}^{2015}$:

$G_{Positive}=\left\{z:D_{P}^{i}>m\cdot \sigma \left(D_{P}\right)\text{,}\;\text{where}\;i=1,2,\ldots ,112\right\}$: group of areas with strong positive tendency;
$G_{Negative}=\left\{z:D_{P}^{i}<-m\cdot \sigma \left(D_{P}\right)\text{,}\;\text{where}\;i=1,2,\ldots ,112\right\}$: group of areas with strong negative tendency;
*G*_*Neutral*_ = {*z*: −*m* ⋅ *σ*(*D*_*P*_) < *D*_*P*_(*z*) < *m* ⋅ *σ*(*D*_*P*_), *i* = 1, 2, …, 112}: group of areas with close to neutral tendency,

where *m* is a certain threshold and *σ*(*D*_*P*_) indicates the standard deviation of *D*_*P*_(*z*).

Additionally we classify the zip codes based on the actual tendency strength, i.e. let us introduce $G_{Positive}^{\prime },G_{Negative}^{\prime },G_{Neutral}^{\prime }$ in the same way as above but replacing the estimated *D*_*P*_(*z*) with the real *D*(*z*) in the corresponding. In this way, compared to defining strong tendency using predicted results, we define strong tendency by the real values and then test the performance of our model by the following indicators.

For each group *G*_*Positive*_, *G*_*Negative*_, *G*_*Neutral*_, we calculate its the normalized population weighted average value of actual *D*(*z*) using the following formulae:

$$\begin{array}{c} {\bar{D}}_{Positive}=\left(\frac{\sum_{i\in G_{Positive}}D\left(z\right)\cdot N\left(z\right)}{\sum_{z=1}^{112}D\left(z\right)\cdot N\left(z\right)}\right)/\sigma \left(D\left(z\right)\right), \end{array}$$

$$\begin{array}{c} {\bar{D}}_{Negative}=\left(\frac{\sum_{i\in G_{Negative}}D\left(z\right)\cdot N\left(z\right)}{\sum_{z=1}^{112}D\left(z\right)\cdot N\left(z\right)}\right)/\sigma \left(D\left(z\right)\right), \end{array}$$

$$\begin{array}{c} {\bar{D}}_{Neutral}=\left(\frac{\sum_{i\in G_{Neutral}}D\left(z\right)\cdot N\left(z\right)}{\sum_{z=1}^{112}D\left(z\right)\cdot N\left(z\right)}\right)/\sigma \left(D\left(z\right)\right), \end{array}$$

where *N*(*z*) is the population of the zip code *z*. Similarly for each of the groups $G_{Positive}^{\prime },G_{Negative}^{\prime },G_{Neutral}^{\prime }$ we calculate the average prediction

$$\begin{array}{c} {\bar{D}}_{Positive}^{\prime }=\left(\frac{\sum_{i\in G_{Positive}}^{\prime }D_{p}\left(z\right)\cdot N\left(z\right)}{\sum_{z=1}^{112}D_{p}\left(z\right)\cdot N\left(z\right)}\right)/\sigma \left(D\left(z\right)\right), \end{array}$$

$$\begin{array}{c} {\bar{D}}_{Negative}^{\prime }=\left(\frac{\sum_{i\in G_{Negative}}^{\prime }D_{p}\left(z\right)\cdot N\left(z\right)}{\sum_{z=1}^{112}D_{p}\left(z\right)\cdot N\left(z\right)}\right)/\sigma \left(D\left(z\right)\right), \end{array}$$

$$\begin{array}{c} {\bar{D}}_{Neutral}^{\prime }=\left(\frac{\sum_{i\in G_{Neutral}}^{\prime }D_{p}\left(z\right)\cdot N\left(z\right)}{\sum_{z=1}^{112}D_{p}\left(z\right)\cdot N\left(z\right)}\right)/\sigma \left(D\left(z\right)\right), \end{array}$$

The values of those quantities for different values of the threshold (*m* = 0.15, example of a very loose threshold classifying most of the predictions as strong, *m* = 0.35, 0.65, 1) are reported in the Tables 7 and 8 and we can see consistent inequalities

$$\begin{array}{c} {\bar{D}}_{Positive}>0>{\bar{D}}_{Negative} \end{array}$$

and

$$\begin{array}{c} {\bar{D}}_{Positive}^{\prime }>0>{\bar{D}}_{Negative}^{\prime } \end{array}$$

holding for all the values of the threshold *m*, which means that our predicted trend directions are consistent with the real trends on average.

**Table 7:** Accuracy of discovering actual strong relative real estate price trends by the predictive model.

Threshold	m = 0.15			m = 0.35		
+/-:Strong Positive/Negative	+	-	Neutral	+	-	Neutral
Number of Observations	23	75	14	20	62	30
${\bar{D}}_{Positive}^{\prime }/{\bar{D}}_{Negative}^{\prime }/{\bar{D}}_{Neutral}^{\prime }$	134.57	-84.28	-3.75	148.60	-95.41	-7.97
Accuracy for Strong P/N	0.7			0.72		
Threshold	m = 0.65			m = 1		
+/-:Strong Positive/Negative	+	-	Neutral	+	-	Neutral
Number of Observations	19	43	50	14	24	74
${\bar{D}}_{Positive}^{\prime }/{\bar{D}}_{Negative}^{\prime }/{\bar{D}}_{Neutral}^{\prime }$	156.73	-114.82	-24.5	179.69	-137.11	-32.56
Accuracy for Strong P/N	0.82			0.77		

**Table 8:** Accuracy of the correspondence of the predicted strong relative real estate price trends to the actual ones.

Threshold	m = 0.15			m = 0.35		
+/-:Strong Positive/Negative	+	-	Neutral	+	-	Neutral
Number of Observations	43	58	11	32	42	38
${\bar{D}}_{Positive}/{\bar{D}}_{Negative}/{\bar{D}}_{Neutral}$	22.61	-75.99	-4.56	42.23	-71.18	-40.78
Accuracy for Strong P/N	0.72			0.77		
Threshold	m = 0.65			m = 1		
+/-:Strong Positive/Negative	+	-	Neutral	+	-	Neutral
Number of Observations	20	31	61	15	12	85
${\bar{D}}_{Positive}/{\bar{D}}_{Negative}/{\bar{D}}_{Neutral}$	44.93	-70.55	-29.83	110.80	-76.29	-41.17
Accuracy for Strong P/N	0.83			0.90		

Moreover, we compare the signs of the predicted values of *D*_*P*_(*z*) for the strong predicted trends *G*_*Positive*_ ∪ *G*_*Negative*_ vs the ground-truth *D*(*z*), as well as the actual values *D*(*z*) for the strong actual trends $G_{Positive}^{\prime }\cup G_{Negative}^{\prime }$, reporting the accuracy ratio of predicting the correct trend direction for strong actual trends and the accuracy ratio for having strong predicted trends to reveal correct trend directions (*D*_*P*_(*z*)*D*(*z*) > 0). Those indicators are listed in Tables 7 and 8 demonstrating the model’s performance.

From Tables 7 and 8, we see that, for around 40 percent of strongest tendency observations or predictions (m = 0.65), our prediction accuracy of a trend direction is higher than 80 percent compared to around 43/62(69%) percent random guess baseline model in Table 7 and 31/51(60.7%) baseline in Table 8. Moreover, in Table 7, we see that when the threshold m increases from 0.15 to 0.65, the accuracy ratio of prediction goes up from 70 percent to 82 percent, meaning that the stronger the actual trend, the more likely to achieve correct prediction. In Table 8, we see that while *m* increases from 0.15 to 1, the accuracy ratio of prediction goes up from 72 percent to 90 percent, hence the stronger the predicted trend, the more accurately our prediction reflects the reality.

The results presented in this section demonstrate that the 311-based model can indeed predict future fluctuations of socio-economic characteristics, including real estate price trends. This serves as an initial proof of concept for multiple potential urban applications using 311 data as a proxy for local socio-economic conditions.

## 7 Discussion

As we see from the results above, 311 service requests can be used in order to characterize the local context of the urban neighborhood and demonstrate moderate to strong correlations with wealth, education level, unemployment, racial structure of its population, as well as with the housing prices in the area. This way 311 data can contribute towards modeling the socio-economic features of the area. Clearly, multiple components of the complex urban systems are strongly related leading to all sorts of correlations between urban characteristics. The fact that the structure of 311 reports is correlated with socio-economic quantities of course does not serve as an evidence of any causal relation—both might simply have the same underlying causes. In addition, 311 reports might pick various causes in different cities, affecting the scalability of the approach. In order to verify the least we applied it to the three different cities—NYC, Boston and Chicago. And while the overall predictive power of 311 service requests is seen in all three, specific patterns/relations are different (also because of the different categorization systems used by 311 in different spatial context). So while modeling of socio-economic characteristics using 311 seems feasible in diverse cities, the models need to be tuned in each specific urban context before they can be applied. Such models could be quite useful for city planners, government, and other urban stakeholders since 311 data is publicly available in nearly real-time compared to official socio-economic measurements collected typically on the annual basis. Of course 311 data has its own limitations. Perhaps, the main one is related to the fact that 311 service requests represent an indirect proxy of the real urban conditions, since propensity to report different types of issues might vary depending on the demographics and other parameters of the location. However, in the current study we aim to verify the modeling/predictive utility of the 311 data as it is, i.e. incorporating both—information of the actual problems and reporting propensity issues at the same time. While the prediction accuracy of socio-economic models based on 311 data exclusively is not perfect and likely insufficient for practical applications, its possible practical utility is two-fold: a) 311-based indicators can strengthen predictive models when used along with other conventional data, b) relative dynamics of 311-based indicators could evidence the underlying dynamics of the socio-economic characteristics of the considered area, enabling early-detection of the local urban trends. The real estate model presented in the previous paragraph, despite its low accuracy, confirms the predictive power of the approach, i.e. its capability of predicting future trends based on the past data in principle.

## 8 Conclusions

A quantitative understanding of urban neighborhoods can be quite challenging for urban planners and policy-makers given significant gaps in the spatial and temporal resolution of data and data collection modalities. However, this subject is crucial for urban planning and decision making, as well as for the study of urban economic and neighborhood change. In this paper, we provide an approach to quantify local signatures of urban function via 311 service request data collected in various cities across the US. These datasets, which can be easily scaled by spatial (zip code, census tracts/block groups, etc.) and temporal level of aggregation, are open to the public and updated regularly. Importantly, we demonstrate consistent relationships between socioeconomic features of urban neighborhoods and their 311 service requests.

For all three cities analyzed—New York City, Boston and Chicago—we demonstrate how clustering of census tracts by the relative frequency vectors of different types of 311 requests reveal distinctive socioeconomic patterns across the city. Moreover, those frequency vectors allow us to train and cross-validate regression models successfully explaining selected socioeconomic features, such as education level, income, unemployment and racial composition of urban neighborhoods. For example, the accuracy of the model explaining local average income in NYC is characterized by a R-squared value of 0.7, while Extra Trees Regression results in a 0.9 out of sample R-squared in explaining housing prices in Chicago (although this must be considered with respect to the smaller sample size). Finally, we illustrate the predictive capacity of the approach by training and validating the model to detect comparative average real estate price trends for zip codes in New York City.

In the nascent field of urban science and more traditional disciplines of economics and urban planning, there is increasing attention on how data collected by cities can be combined with novel machine learning approaches to yield insight for researchers and policy-makers. It is possible that such data can be used to better understand the dynamics of local areas in cities, and support more informed decision-making. In addition, it is conceivable that a set of efficient instrumental variables based on widely-available 311 data can be used to replace survey-based socioeconomic statistics at spatio-temporal scale where such official survey data is non-existent or inconsistent, thus broadening opportunities for urban analytics.

## Supporting information

S1 Text Unsupervised model and cluster number selection. (PDF)

S2 Text Classification result based on 311 service requests timeline data. (PDF)

S3 Text Model selection, cross-validation and out of sample R-squared. (PDF)

S4 Text Choice of scale among zip code, census tract and census block. (PDF)

S5 Text Spatial autocorrelation in 311 patterns. (PDF)

S6 Text Clustering with outlier detection. (PDF)

S7 Text Clustering with spatial regularization. (PDF)

S1 Fig Elbow method. (EPS)

S2 Fig Silhouette score. (EPS)

S3 Fig Classification of urban locations based on the timeline data of 311 request services. (EPS)

S4 Fig Hourly distribution of 311 service requests among clusters. (EPS)

S5 Fig Number of areas vs request activity per area. (EPS)

S6 Fig Distributions of autocorrelation values for different categories of requests (Moran’s I). (EPS)

S7 Fig DBSCAN clustering for New York. (EPS)

S8 Fig KMean clustering with spatial regularization. (TIF)
