# Supplementary material for: Structure of 311 service requests as a signature of urban location
Source: PLoS One. 2017 Oct 17;12(10):e0186314. doi: 10.1371/journal.pone.0186314 (PMC5645100; doi:10.1371/journal.pone.0186314)
Supplement: S1 Text — (PDF) [file pone.0186314.s001.pdf]

Supplementary Text 1. Cluster number selection

Let S be a set of observations (locations in our data set), and a clustering U on S is a way of partitioning S into non-overlapping subsets $U_1, U_2, ..., U_k$. We will investigate how well the model performs with different number of clusters, i.e. different k.

Here we choose K-means clustering with four clusters as our basic model. We made this decision based on the two clustering evaluation methods: Silhouette method and Elbow method[1].

S1.1 Silhouette method

Silhouette is a commonly used method of interpretation and validation of consistency within clusters of data. It was first described by Peter J. Rousseeuw in 1986[3] and it measures how similar an object is to its own cluster (internal relation) compared to other clusters (external relation). The Silhouette score ranges from -1 to 1, where higher value indicates better match to its own cluster and, at the same time, poorer matched to neighboring clusters—hence, higher Silhouette score means a better model overall as it highlights the distinctions among clusters.

The Silhouette value can be calculated with any distance metric, such as the Euclidean distance we applied here. We have run the tests for all three cities. The results are plotted on Fig. S2. We can see that:

- New York City: Models with 2, 3, and 4 clusters seem closely comparable and outperform the rest;
- Boston: Models with 2 and 4 clusters have the highest Silhouette scores;
- Chicago: Silhouette score appears to be decreasing as the number of clusters rises, the optimal choice is 2.

Figure S2. Silhouette method

This observation tells us two things:

- Models with 2, 3, and 4 clusters are generally better than others;
- 2-cluster model seems to be the best choice in terms of Silhouette's quantitative criteria.

Next we try Elbow method, another validation approach described in next subsection, before making final decisions.

S1.2 Elbow method

The Elbow method measures how "cost-efficient" a model is by looking at the percentage of variance explained as a function of the number of clusters. It searches for a balance between "more information" and "less complicated model". Intuitively, if we start from 1-cluster model (which is no processing at all, just leave them as a whole), adding another cluster should give more information about the data distinction, but one should stop when the marginal gain is insignificant compared to the cost. Then the number of clusters is chosen at this point[5].

Equivalently, we can check the average sum of squared errors. Of course, we want our error as small as possible, and the error tends to decrease toward 0 as we increase the cluster number, k (the error is 0 when k is equal to the number of data points in the dataset, because then each data point is its own

Table S1. Optimal choices based on each evaluation method

City	Silhouette	Elbow
NYC	3	4
Chicago	2	3, 4
Boston	2, 4	2

cluster, and there is no error between this point and the center of its cluster–that point itself). The goal is the same: search for the point where the marginal drop is no longer attractive beyond it.

The results are summarized in Fig. S1 :

- New York City: Obviously the error drops rapidly before 4 and then slows down after 4, so 4-cluster model is the best choice here;
- Chicago: Very similar to NYC, although the change is a bit mild and both - 3 and 4 - seem to be good choices;
- Boston: The trend does not provide any intuitive number of clusters to focus on.

Figure S1. Elbow method

S1.3 Conclusion

To sum up, we have the following observations among three major cities in Table S1:

Since 4 is the only number that has appeared in all three rows, and clearly 4 clusters can reveal more details about the city structures than 2 or 3, we think that 4-cluster model may the best overall choice. Choosing 4 instead of, say, 2, in our opinion, is a reasonable trade-off between having more clusters and still decent clustering quality.

References

1. MacQueen J. Some methods for classification and analysis of multivariate observations. Proceedings of the Fifth Berkeley Symposium on Mathematical Statistics and Probability. 1967;1: Statistics: 281–297.
2. Vinh, N Xuan and Epps, Julien. Information Theoretic Measures for Clusterings Comparison: Variants, Properties, Normalization and Correction for Chance. Journal of Machine Learning Research 2010; 11:2837-2854
3. Rousseeuw P. Silhouettes: A graphical aid to the interpretation and validation of cluster analysis. Journal of Computational and Applied Mathematics. 1987;20: 53–65.
4. Kaufman L, Rousseeuw P. Clustering by means of medoids. In: Dodge Y, editor. Statistical Data Analysis Based on the L1 Norm and Related Methods. North-Holland; 1987. p. 405–416.
5. Ketchen, J. David and Shook, L. Christopher. The application of cluster analysis in Strategic Management Research: An analysis and critique. Strategic Management Journal 1996; 17 (6): 441458. doi:10.1002/(SICI)1097-0266(199606)17:6¡441::AID-SMJ819¿3.0.CO;2-G.
