# Supplementary material for: Structure of 311 service requests as a signature of urban location
Source: PLoS One. 2017 Oct 17;12(10):e0186314. doi: 10.1371/journal.pone.0186314 (PMC5645100; doi:10.1371/journal.pone.0186314)
Supplement: S2 Text — (PDF) [file pone.0186314.s002.pdf]

Supplementary text 2. Classification result based on 311 service requests timeline data

The 311 service request data is pretty rich and although types of requests considered in the paper provide an important and useful perspective for spatial clustering and modeling socio-economic quantities, there are other interesting dimensions in the data to consider. In this supplementary paragraph, we provide an alternative approach to conduct the clustering analysis based on 311 service requests data. Instead of using types of 311 service requests, we consider their timeline, building our new data set by accumulating all types of 311 services requests during each hour of the week for each zip code area. Thus this new data set includes 168 features (activity distribution per hours within an average), for all the zip code areas within New York City.

Based on the new 168 dimensional feature space, we divide the zip code areas in NYC into four clusters using K-Means clustering algorithm, highlighting different temporal patterns in 311 service request activity. The clustering result is shown on the Fig. S3 Fig, while the corresponding 311 service request timelines for different clusters — on the Fig. S4 Fig.

Figure S3 Fig. Classification of urban locations based on the timeline data of 311 request services

Figure S4 Fig. Hourly distribution of 311 service requests among clusters.

Figure S2.2 shows that the timelines of 311 service requests among Clusters 1,2,3 are rather similar. But Cluster 4 can be distinguished since the service requests have a considerable spike after 8 PM each day and especially over the weekends, indicating evening-time activity in those areas, which largely include locations across Manhattan, which makes common sense. Further understanding this pattern might require additional analysis of the service requests' types and other contextual information. However the overall difference between socio-economic factors among different clusters is much less significant than the result based on 311 service request type data shown in Fig. 4 in the main text.

Therefore, for the purpose of the socio-economic analysis of this study we decided to stick to the service request types other the timeline.
