# Supplementary material for: Structure of 311 service requests as a signature of urban location
Source: PLoS One. 2017 Oct 17;12(10):e0186314. doi: 10.1371/journal.pone.0186314 (PMC5645100; doi:10.1371/journal.pone.0186314)
Supplement: S3 Text — (PDF) [file pone.0186314.s003.pdf]

Supplementary Test 3. Model selection, Cross-validation and Out of Sample R-squared

The results of the model selection are shown in Table 2. The following procedures are applied for each city.

Assuming we have data set X and labels y, where X is $n \times m$ matrix and y is $n \times 1$ vector. All the entries are real values.

Firstly, we collect all the possible models for training. The types of the models we consider include: Lasso, Neural Networks with regularization, Random Forest Regression, and Extra Trees Regression. We treat models with different hyper-parameters set as different models even they are belong to the same model type.

Secondly, we randomly split the whole data set into training data and test data. We train each model's parameters on training set, and get the out of sample R-squared from the prediction result on testing set. We repeat this process ten times, and record the average R-squared for each model.

Finally, we report the largest out of sample R-squared among all the records(models) from second part and write it in Table 2.

References

1. Tibshirani, Robert. Regression Shrinkage and Selection via the lasso Journal of the Royal Statistical Society. Series B (methodological) 1996. 58 (1). Wiley: 26788.

2. Girosi, Federico; Michael Jones; Tomaso Poggio. Regularization Theory and Neural Networks Architectures Neural Computation 1995. 7 (2): 219269.

3. L.Breiman Random Forests Machine Learning, 45(1), 5-32, 2001.
