# Supplementary material for: Structure of 311 service requests as a signature of urban location
Source: PLoS One. 2017 Oct 17;12(10):e0186314. doi: 10.1371/journal.pone.0186314 (PMC5645100; doi:10.1371/journal.pone.0186314)
Supplement: S4 Text — (PDF) [file pone.0186314.s004.pdf]

Supplementary Text 4. Choice of scale among zip code, census tract and census block.

Consider the spatial granularity for the spatial aggregation of the New York City's 311 service requests, choosing among the three options: zip code areas, census tract areas, and census block areas of New York City.

The primary goal for this scale selection is to find the right balance between the number of spatial units which will serve as observations for our model and the sparsity of the data. In Fig. S4, we use x-axis for the number of total requests in each area (zip code level, census block level, etc), and for each given x show the number y of areas with request activity higher than x. We hope to find an appropriate scale such that it provides both adequate number of areas to analyze and abundant request activities to analyze per each area.

- Let's start with Zip Code scale–only 178 total observations at hand, it's too few to apply various machine learning algorithms for our research, despite most zip code areas have more than 500 activities in total.
- Census Block scale, on the other hand, offers more than 6000 areas in total. But for most of these areas (93%), total activities are less than 500.
- In comparison, Census Tract data set has 1367 observations with more than 500 requests, which seems to be a good balance addressing the issue of data sparsity as well as providing enough areas to analyze.

Hence we have selected Census Tract as the basic spatial scale for our research.

Figure S4. Number of areas vs Request Activity per Area
