# Supplementary material for: Structure of 311 service requests as a signature of urban location
Source: PLoS One. 2017 Oct 17;12(10):e0186314. doi: 10.1371/journal.pone.0186314 (PMC5645100; doi:10.1371/journal.pone.0186314)
Supplement: S5 Text — (PDF) [file pone.0186314.s005.pdf]

Supplementary Text 5. Spatial autocorrelation in 311 patterns

As we discussed in the main text, 311 records exhibit certain level of spatial autocorrelation. Most of the 311 request categories demonstrate modest spatial autocorrelation, while some, such as Fire Safety Inspection Complaints for New York, Graffiti and Abandoned Buildings for Chicago and Boston, show a more substantial one. The distributions for each city are reported on Fig. S6.

Figure S6. 311 Complains Autocorrelation (Moran's I) Distributions
