# Supplementary material for: Structure of 311 service requests as a signature of urban location
Source: PLoS One. 2017 Oct 17;12(10):e0186314. doi: 10.1371/journal.pone.0186314 (PMC5645100; doi:10.1371/journal.pone.0186314)
Supplement: S6 Text — (PDF) [file pone.0186314.s006.pdf]

Supplementary Text 6. Clustering with outlier detection

K-means clustering of the 311 patterns provide meaningful and consistent clusters, however certain level of noise is present in the resulting spatial pattern. Now, we apply a different algorithm, DBSCAN [Ester, Martin, et al. "A density-based algorithm for discovering clusters in large spatial databases with noise." Kdd. Vol. 96. No. 34. 1996.] able to deal with the outliers. Features below the distance threshold are dropped as noise. As displayed on Fig. S6, DBSCAN picked 4 clusters, basically outlining the cores of the regions we've seen in k-means version.

Figure S6. DBSCAN clustering results for New York
