# Supplementary material for: Structure of 311 service requests as a signature of urban location
Source: PLoS One. 2017 Oct 17;12(10):e0186314. doi: 10.1371/journal.pone.0186314 (PMC5645100; doi:10.1371/journal.pone.0186314)
Supplement: S7 Text — (PDF) [file pone.0186314.s007.pdf]

Supplementary Text 7. Clustering with spatial regularization

In the section 2 we apply k-means clustering to the 311 patterns without any spatial considerations taken into account. Still, the results are spatially cohesive.

Here we add spatial regularization for comparison. Specifically for each census block group, we add a pair of centroid coordinates X and Y in the state plane coordinate system to the vector characterizing the 311 pattern. The coordinates are normalized by a multiplicative coefficient ensuring that average geospatial distance is equal to the overall 311-related distance. Then an additional weight coefficient is introduced to X,Y controlling the relative weight of the X,Y in comparison with the 311 pattern. On Fig. S6 we display clustering with different weights for spatial regularization. As expected, with the increase of the spatial weight, clusters start to be more spatially cohesive; Southern Brooklyn merges with Staten Island, and Northern Brooklyn with Queens. Still, strong ties between Bedford-Stuyvesant area and Bronx remain. In general spatial regularization removes noise and spatial outliers, thus it might help urban planning and zoning applications of the 311-based analysis.

Figure S6. KMean Clustering with Spatial Regularization of different weights.
