# Supplementary figures and images for: Structure of 311 service requests as a signature of urban location
Source: PLoS One. 2017 Oct 17;12(10):e0186314. doi: 10.1371/journal.pone.0186314 (PMC5645100; doi:10.1371/journal.pone.0186314)

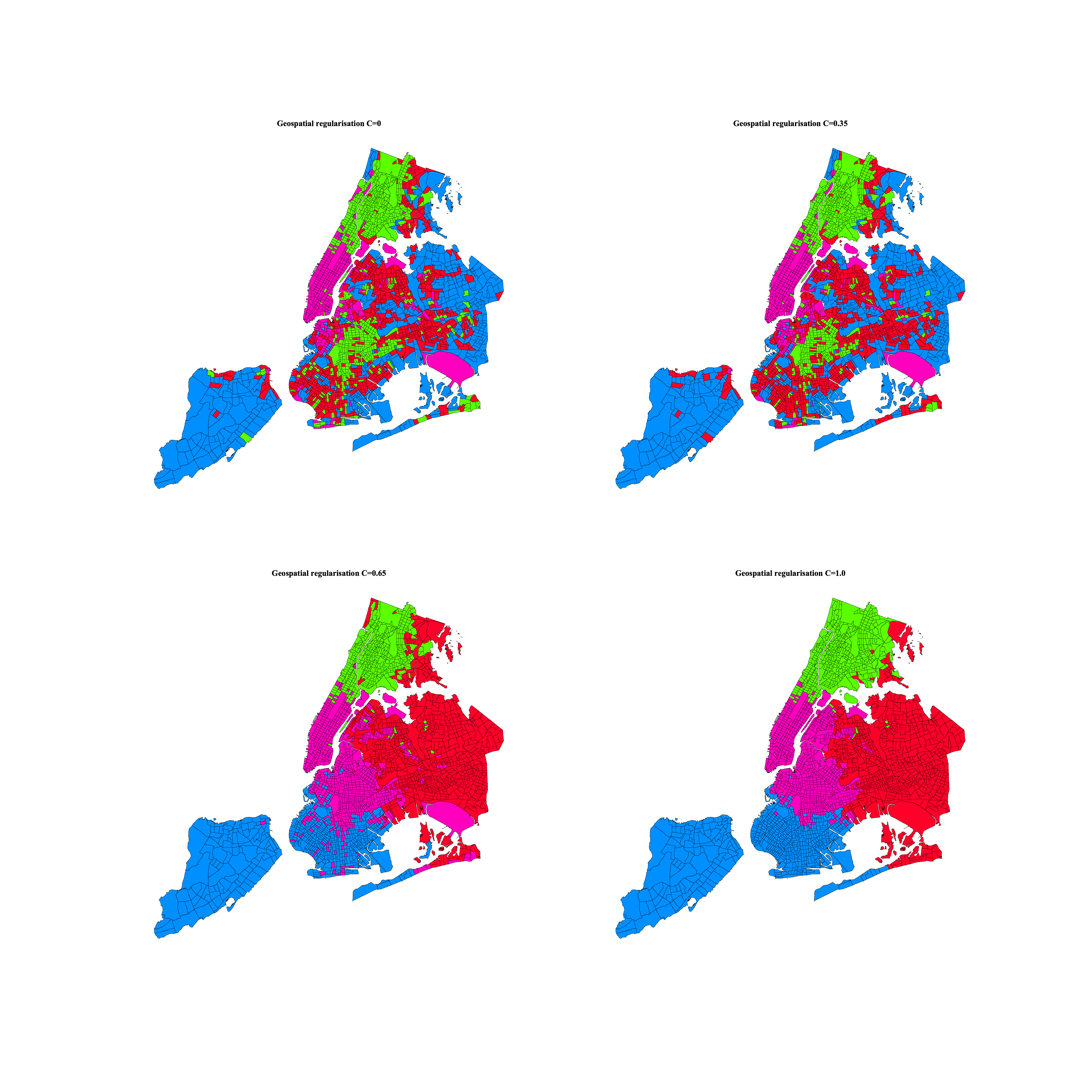

Supplement: S8 Fig — (TIF) [file pone.0186314.s015.tif]
